# Implementation and evaluation of the 5As framework of obesity management in primary care: design of the 5As Team (5AsT) randomized control trial

**DOI:** 10.1186/1748-5908-9-78

**Published:** 2014-06-19

**Authors:** Denise L Campbell-Scherer, Jodie Asselin, Adedayo M Osunlana, Sheri Fielding, Robin Anderson, Christian F Rueda-Clausen, Jeffrey A Johnson, Ayodele A Ogunleye, Andrew Cave, Donna Manca, Arya M Sharma

**Affiliations:** 1 Department of Family Medicine, Clinical Research Unit, University of Alberta, 2-004 Li Ka Shing Ctr, 87 Avenue and 112st, Edmonton, AB T6G 2E1, Canada; 2 Department of Medicine, Obesity Research & Management, University of Alberta, Li Ka Shing Building, Rm. 1-116, 87th Avenue and 112th Street, Edmonton, AB T6G 2E1, Canada; 3 Edmonton Southside Primary Care Network, Suite 200, 9808-42 Avenue, Edmonton, AB T6E 5V5, Canada; 4 School of Public Health, University of Alberta, 2–040, Li Ka Shing Center for Health Research Innovation, Edmonton, AB, Canada

**Keywords:** Primary healthcare, Obesity, Randomized control trial, Evaluation studies, Family medicine, Practice facilitation

## Abstract

**Background:**

Obesity is a pressing public health concern, which frequently presents in primary care. With the explosive obesity epidemic, there is an urgent need to maximize effective management in primary care. The 5As of Obesity Management™ (5As) are a collection of knowledge tools developed by the Canadian Obesity Network. Low rates of obesity management visits in primary care suggest provider behaviour may be an important variable. The goal of the present study is to increase frequency and quality of obesity management in primary care using the 5As Team (5AsT) intervention to change provider behaviour.

**Methods/design:**

The 5AsT trial is a theoretically informed, pragmatic randomized controlled trial with mixed methods evaluation. Clinic-based multidisciplinary teams (RN/NP, mental health, dietitians) will be randomized to control or the 5AsT intervention group, to participate in biweekly learning collaborative sessions supported by internal and external practice facilitation. The learning collaborative content addresses provider-identified barriers to effective obesity management in primary care. Evidence-based shared decision making tools will be co-developed and iteratively tested by practitioners. Evaluation will be informed by the RE-AIM framework. The primary outcome measure, to which participants are blinded, is number of weight management visits/full-time equivalent (FTE) position. Patient-level outcomes will also be assessed, through a longitudinal cohort study of patients from randomized practices. Patient outcomes include clinical (*e.g*., body mass index [BMI], blood pressure), health-related quality of life (SF-12, EQ5D), and satisfaction with care. Qualitative data collected from providers and patients will be evaluated using thematic analysis to understand the context, implementation and effectiveness of the 5AsT program.

**Discussion:**

The 5AsT trial will provide a wide range of insights into current practices, knowledge gaps and barriers that limit obesity management in primary practice. The use of existing resources, collaborative design, practice facilitation, and integrated feedback loops cultivate an applicable, adaptable and sustainable approach to increasing the quantity and quality of weight management visits in primary care.

**Trial registration:**

NCT01967797.

## Background

Obesity is a common problem in primary care [1,2]. There are substantial direct and indirect costs to the healthcare system; conservative estimate of costs attributable to obesity in Alberta in 2005 totaled $1.27 billion [3]. Studies suggest that a primary care-based obesity treatment model could be cost-effective over the long term [2] and that treating obesity can reduce the incidence of a variety of chronic diseases [4-7]. However, obesity is ‘not effectively managed within our current primary health system’ [4-6]. To address this problem, a tool for obesity counseling and management in primary care settings, known as the 5As of Obesity Management™ has been developed [7]. This tool incorporates the conceptual structure of the Best Practices in Weight Management document, the Canadian Obesity Clinical Practice Guidelines [8], and the 5As methodological framework (Ask, Assess, Advise, Agree, Assist) [9]. Preliminary evidence shows that use of the 5As of Obesity Management can increase provider-client interactions in weight management [10]. However, the 5As have not been evaluated in a system-wide implementation study.

The Primary Care Network (PCN) model in Alberta has a 10-year history of embedding multidisciplinary teams in pre-existing family physician clinics. Chronic disease nurses and nurse practitioners working in this multi-disciplinary setting (with family physicians, mental health workers and dieticians) present a good model to target and assess improvement in obesity management.

The goal of this project is to implement and evaluate the 5AsT team intervention aimed at changing provider behaviour with regard to obesity management. This intervention, informed by the theoretical domains framework for behaviour change and the conceptual framework of complex innovation implementation, is co-developed with end-users, emphasizing bidirectional knowledge transfer among multidisciplinary team members to develop a pragmatic and sustainable approach to obesity management in primary care.

## Methods

### 5AsT study overview

The 5AsT trial is a theoretically informed, pragmatic randomized controlled trial with convergent mixed methods evaluation of an intervention on primary care providers to improve obesity management. Clinic-based multidisciplinary teams (RN/NP, mental health, dieticians) will be randomized to control or the 5AsT intervention group. The intervention providers will participate in biweekly learning collaborative sessions supported by internal and external practice facilitation. These learning collaboratives will explore provider-identified barriers to effective weight management in primary care. Evidence-based shared decision making tools will be co-developed and iteratively tested by practitioners. The primary outcome measure is the number of weight management visits per full-time equivalent (FTE) RN/NP position. This measure is longstanding, routine administrative data in the PCN. Participants are unaware of the primary outcome measure, and the research team is blinded to the result during the study period. Patient-level outcomes will be assessed, through a longitudinal cohort study of patients from randomized practices. Qualitative data will be collected from providers and patients, and evaluated using thematic analysis to understand the context, implementation and the effectiveness of the 5AsT intervention. Patient-level outcomes including clinical, health-related quality of life, and satisfaction with care will also be assessed.

**Figure 1:**
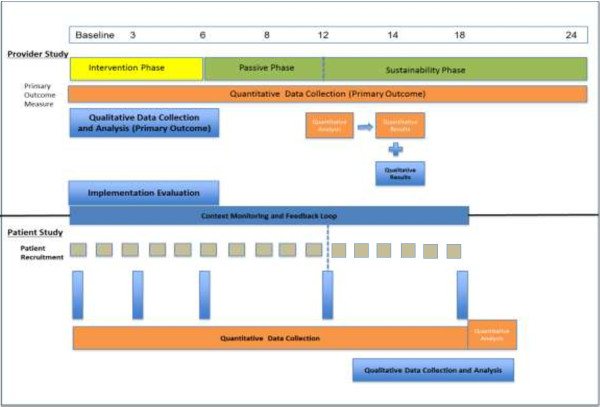
**5AsT study overview.** The upper portion pertains to the provider-level study and shows intervention and evaluation timeline. The lower portion pertains to the parallel patient-level study.

Figure 1 provides an overview of the 5AsT trial, which consists of both a provider-level intervention study and a patient-level impact assessment. The 5AsT provider-level study is divided into three phases. Phase 1 is the ‘Intervention Phase,’ which consists of a kick-off session followed by bi-weekly two-hour learning collaborative sessions over six months. Phase 2 is the ‘Passive Phase,’ a six-month period where we provide no direct support to the 5As Teams but continue to collect data to determine if behavior change has been internalized. Phase 3 is the ‘sustainability phase,’ where the primary outcome measure continues to be collected over 12 months to determine if change can be sustained over time. The patient-level study will assess how patients coming from 5AsT intervention practices engage in weight management efforts over time compared to patients from control practices.

### Guiding theoretical frameworks

#### Conceptual framework of complex innovation implementation

Complex innovations such as behavioral change interventions in primary care can be conceived using this validated framework [11]. Our alignment with this framework reduces the chance that context change will negatively affect implementation or completion of the project. This framework informed the decision to have a clinical champion act as an internal practice facilitator from within the primary care network.

### Theoretical domains framework for behaviour change

The Theoretical Domains Framework is a validated, comprehensive overview of the core domains important to consider in behavioural change interventions in healthcare improvement [4]. This Framework informs our intervention on the practitioners to expand from knowledge alone, towards engaging in all components critical for their behaviour change. The 5AsT intervention leverages the clinical champion role for coaching and, the practice facilitation role to aid in logistical support, and the learning collaborative model to increase role identification, goal setting, identification of barriers and facilitators to action. We also used this framework to determine the target patient population. The patient study focuses on patients who have committed to action prior to recruitment (‘activated patients’), assessing their ability to initiate and sustain weight management efforts.

### Guiding evaluation framework: RE-AIM

Our overall evaluation summarized in Table 1, is guided by the RE-AIM framework: Reach into the target population; Effectiveness of the intervention; Adoption by target settings; Implementation including consistency and cost of delivery; and Maintenance of intervention effects over time (sustainability) [5,6,12,13].

**Table 1:** RE-AIM framework as a guide for project evaluation

**Domain**	**Description**	**Measure for patients**	**Measure for providers**
**R**each	Degree to which target population is reached	• number recruited	• Control/intervention groups
		• percent attrition	• Intervention attendance
		• patient characteristics	• Provider-chosen topics (subject appropriateness)
**E**ffectiveness	Impact on study outcome	• SF 12	• Quantitative primary
		• BMI	outcome measure
		• 5AsT vs. non 5AsT patients	• Self-reported efficacy
**A**daptation	Organizational uptake	Not applicable	• Sustainability phase
			• Repeat provider interviews
**I**mplementation	Intervention implementation as intended	Not applicable	• Learning collaborative
			• Organizational by-in
			• Practice facilitation
			• Feedback loops
**M**aintenance	Can program outcomes be sustained over time?	• Longitudinal data collection	• Longitudinal data collection

### Setting/population

The 5AsT intervention was designed with our partner primary care network (PCN), which consists of 59 dedicated multidisciplinary healthcare providers (nurses, nurse practitioners, mental health workers, dieticians, exercise physiologists, respiratory therapists) embedded in 46 family practices with over 160 family physicians serving 192 655 Albertans. The PCN has been in a period of rapid growth.

At the practice level, the RN/NPs in the PCN are responsible for significant chronic disease management, including diabetes and weight management, as well as prenatal care.

To be eligible to be randomized to the intervention, PCN-affiliated family practice clinics must have joined the PCN by April 2013, and must have a multidisciplinary team including a nurse/nurse practitioner, mental health worker and a dietician affiliated with the clinic, resulting in 24 eligible clinic teams. The intervention unit is the PCN multidisciplinary team affiliated with the clinic, referred to as the ‘5AsT team.’

Patients are eligible to be recruited to the longitudinal cohort study if they declare activation for behaviour change through their enrolment and participation in one of the PCN programs for health (*e.g*., weight management groups, activity groups, mental health groups). These are run independently from the clinic-based 5AsT team. Patients are eligible regardless of what clinic they attend for their regular care.

### Intervention and control

The 5AsT intervention builds upon the knowledge product of the 5As of Obesity Management™ and extends it to a pragmatic, practice-based intervention for provider behaviour change. The 5AsT intervention will occur in biweekly learning collaborative sessions for six months. The content of the 5AsT learning collaborative sessions will be determined with the practitioners randomized to the intervention. The 5As teams will be supported by a 5As Champion, a recognized clinical leader in weight management from the PCN. This individual is identified and remunerated through the PCN and functions as an internal practice facilitator for the project. In addition to assisting the research team with coordinating their actions with the clinical operations of the PCN, the Clinical Champion serves as a coach and mentor to the 5AsT team members, and as a facilitator for the learning collaborative. Additional support to the 5AsT teams is provided by two external practice facilitators who identify resources, design prototype tools, collect feedback and coordinate with content experts, physicians, and graphic designers to refine the 5AsT tools.

Providers randomized to the control arm of the study all receive usual professional development courses for obesity management through Alberta Health Services and the PCN, which includes didactic training on the 5As of Obesity Management tool kit. Control providers will not take part in bi-weekly learning collaboratives, will not be given circulated learning materials, and will not receive added support from practice facilitators.

### Provider-level study

To test the effect of the 5AsT intervention, we will conduct a pragmatic, mixed methods, allocation concealed, randomized, blinded (outcome, data analysts), clinical trial. Figure 1 (upper) details the provider-level study.

### Hypotheses

1. Implementation of the 5AsT in primary care practice will increase the number of weight management visits per FTE conducted by the PCN RN/NPs. The primary outcome measure is the number of weight management visits as a function of provider full time equivalent (FTE) work (*i.e*., a half time nurse has an FTE of 0.5).

2. Implementation of the 5AsT in primary care practice will result in sustained changes in medical practice as evaluated by the RE-AIM framework [13,14].

### Qualitative primary question

What contextual factors affect the number of weight management visits conducted by the PCN practitioners?

### Allocation concealment and randomization

Following ethical approval and study registration, the eligible clinics (N = 24) were allocation concealed and randomized in a 1:1 ratio using a computer generated random sequence by a statistician external to the project. Randomization was stratified for larger vs. smaller patient panel sizes. Three strata with eight practices were created from the 24 eligible practice units. The first 8 units (Group 1) have panel size ≤2,754; Group 2 has clinic panel size from 2,755 to 6,576, and Group 3 has clinic panel size ≥6,577.

### Outcome ascertainment, blinding and equal treatment

The primary outcome measure is the number of weight management visits conducted by the RN/NP participants in their individual practices. Their practice involves many different kinds of clinical activities like prenatal visits, diabetes care, and other chronic disease management visits.

The primary outcome measure is a routine measure of clinical activity collected on standardized forms within the PCN at every clinical encounter; there is already an audit and quality assurance process in place for this measure. Due to the fact some provider participants work part time, or fractional FTE (full-time equivalent), this must be included in the primary outcome measure.

The provider subjects will be blinded to knowledge of the primary outcome measure so as not to influence their behavior in data collection. The research team will not be involved with the collection of the primary outcome measure. The investigators and health practitioners will have no access to this data and will remain blinded to the results until 12 months following implementation. Data analysts will remain blinded to the allocation of intervention versus control.

Aside from the 5AsT intervention programming, there is no difference in treatment between the intervention and control practitioners. The RN/NPs’ clinical practices are geographically dispersed and do not routinely interact.

### Quantitative statistical analysis plan

The data will be stored in PCN clinical database and will be extracted after the 12-month intervention and passive period. We will assess the outcome at baseline, 3-month, 6-month, and 12-month time points. The data will be analyzed for multiple time points to allow for a comparison between immediate and long-term provider impact and to increase the reliability of observed trends. Following the sustainability period of 12 to 24 months, the data will be extracted again, and analyzed by blinded data analysts.

### Primary outcomes

Two stage summary statistics will be used for the analysis of the primary outcome; the number of weight management visits/FTE for each practitioner will be derived for each time point, and the average of the weight management visits/FTE for the intervention and the control group will be calculated. Weight management visit/FTE trends at pre-intervention, baseline, 3, 6, 12 and 18 months post intervention will be plotted to compare the two groups.

The intervention group and control group will be compared using Wilcoxon-Mann–Whitney test. As generalized estimating equation (GEE) can adjust for clustering effect and does not require a normal distribution, we will perform GEE models to compare the 5AsT intervention group and the control group for our primary outcome at 3, 6, 12 and 18 months. Analysis will be by intention to treat.

### Power considerations

Power calculations were performed using both simple and cluster randomization where each clinic is considered as a cluster and RNs are clustered within units. The intra class correlation was estimated to be 0.40. Power calculations with the two approaches were very similar. Given the large numbers of units with only one nurse, we opted in favor of a simple randomization approach. Briefly, this initial approach was as follows:

For simple randomization, a power of 77% was estimated from N = 31 (total number of nurses in the study). The power for a clustered randomized trial was estimated for two scenarios: in 24 clinics with an average of two nurses per clinic, this resulted in a power of 80%; in 24 clinics with an average of one nurse per clinic, it resulted in a power of 65%. These two were presented because we have 31 nurses in total, with an average of 1.3 nurses per clinic, and the exact calculation is not available because an integer is required. However, from the two scenarios, the exact power should be somewhere between 65% and 80%, which is similar to the resulting power from a simple randomization (77%), thus explaining our rationale of opting for a simple randomization.

Effect size was determined using the 22 units for which complete baseline data was available. The mean number of weight management visits/FTE was 69.2 with a standard deviation of 48.1. The study will have 80% power to detect an effect size of 1.19 (absolute difference of 57-weight management visits/FTE between intervention and control groups).

### Qualitative data collection

Qualitative data will include description of context, implementation process, and effect of the 5AsT intervention on provider behaviour change. The approach is summarized in Table 2. Primary data sources for the intervention include guided field notes taken during bi-weekly learning collaborative sessions, and logs kept by the clinical champion and the practice facilitators. Data sources for provider impact include semi-structured interviews with key informants, and focus groups. Potential participants will include all providers and key PCN implementation personnel involved in the 5As Team project.

**Table 2:** Qualitative data collection plan

**Method**	**Justification**	**Timeframe**
**Intervention Phase**		
Session field notes	Description: context, implementation process.	0-6 months
Semi-Structured Interviews	All 5AsT randomized providers. Baseline data: intervention content and process feedback-loop.	Initial 3 months
	Personal views and practice, values fit, clinic climate.	
Focus Groups	Evaluation of tools developed during sessions.	6 months
**Passive Phase**		
Log book Diary notes of passive observations on clinical impact. 0–12 months Clinical Champion		
**Sustainability Phase**		
Focus Groups	Best practices and intervention impact during the passive phase.	12-24 months
**Data Mixing**		
Semi-structured interviews with key providers.	Follow-up of emergent questions.	14-16 months
Semi-Structured interviews with selected patients.	Contextual factors that may have influenced patient behaviors.	18-24 months

### Qualitative data handling and analysis

Analysis of qualitative data will continue throughout the project. Immediately following each observed intervention session, observers will meet at the PCN and construct a composite field note of the event directly into one of the team computers. Field notes will be entered into NVIVO 10 software (QSR International, Burlington, Mass.) Interviews will be audio recorded, transcribed verbatim, and entered into NVIVO.

Thematic Analysis will be the primary qualitative analysis approach for this project [15-18]. Thematic analysis refers to the systematic search for and identification of common themes that are present in data (transcripts and field notes). The unit of analysis will be the healthcare practitioner. Inductive rather than predetermined coding was chosen in order to allow themes to emerge from the data itself and reflects an exploratory rather than explanatory approach.

All interview transcripts will be coded and compared by more than one individual to ensure reliability. A coding manual will clearly outline code definitions and use. A clear record of how themes were generated from raw data will be reviewed by all team members.

### Qualitative and quantitative data mixing

Qualitative data analysis will be conducted on an iterative base and informs the intervention. The quantitative data for the primary outcome measure, number of weight management visits/FTE, will be collected in a blinded fashion for the first 12 months. At this point, the primary outcome measure will be analyzed. The study team will then be un-blinded and results will be compared with those from the qualitative analysis. It is expected that themes emerging from qualitative data will be reflected in patterns of quantitative data. Any correlation, or lack thereof, will be explored using key informant semi-structured interviews and focus groups. The purpose of this parallel mixed methods design is fourfold: first, to avoid bias during qualitative analysis; second, to explain any variability of the primary outcome measure; third, to record elements of RE-AIM not captured in the quantitative measures for the different providers in the intervention group; and fourth, to monitor the impact of context, and implementation process in part allowing for real-time feedback loops to maximize effective implementation.

### Patient-level study

The 5AsT patient portion of the study occurs concurrently with the provider study. Figure 1 (lower) details the patient-level study.

### Hypotheses

1. Implementation of the 5AsT in primary care, in addition to PCN weight management programs, will improve patient important outcomes: primary measures (weight, body mass index [BMI], Short Form 12-item Health Survey [SF-12]) and secondary measures (blood pressure [BP], waist circumference [WC], EuroQol EQ-5D [EQ-5D^TM^], modified Patient Assessment of Chronic Illness Care [PACIC] [19-22] and completion of recommended biomedical testing for those age > 40 or diabetic).

2. Activated patients, as defined as those who have elected to participate in PCN programming for weight management, will have improvement in patient-important outcomes with PCN programming.

3. Patients who attend 5AsT intervention practices will see improved sustained results greater than in those who attend practices that have standard PCN programming alone.

### Qualitative primary question

What contextual factors affect patient perception of in-clinic weight management efforts?

### Subject recruitment

A key feature of pragmatic trials is that the participants reflect the population for which the treatment is intended. For the widest generalizability, it is therefore essential that exclusion criteria be kept to a minimum. Inclusion criteria will be all adult patients older than 18 years with a BMI ≥25, enrolling in PCN programs for health, able and willing to give written informed consent in English. Children and pregnant women will be excluded. Since this is a trial of the primary care management of obesity, patients whose obesity is co-managed by an obesity specialist or tertiary care center will also be excluded (*e.g*., patients referred for bariatric surgery), as well as patients who are unable to participate in regular clinic visits or programs due to geographic, social or physical reasons.

### Power considerations

The sample size calculation for the patient cohort study is powered based upon the SF-12 and the BMI. For SF-12, a moderate effect size is 0.3, and for a 5% reduction in BMI, a moderate effect size is 0.23 to 0.25. We will aim for 80% power. We will gear our enrolment goal to anticipate a 30% lack of adherence to the complete measurement protocol, ensuring that in this scenario the power will remain reasonable at 70%.

### Procedures: enrollment and data collection visits

Due to ethical and logistical considerations, we were not permitted to randomize directly at the patient level. Thus, we have randomized at the clinic level. We cannot control how many patients enter the evaluation from each clinic, but based on baseline practice size data, we anticipate that there should be balanced representation from 5AsT and control clinics.

Activated patients will be approached for recruitment into the study. Signed informed consent will be obtained at the PCN by trained staff. Patient visits will occur at baseline, 3, 6, 12 and 18 months at the PCN. We will endeavour to have a minimum of 6 months of data on all patients; thus recruitment must end in October 2015 to close the study by end of March 2016. Proposed baseline characteristics of the patients will also be included (Table 3).

**Table 3:** Demographic characteristics and health variables to be collected on patients

Age in years (mean ± SD)	BMI (mean ±SD)
Gender (% female)	Weight status (%):
Ethnic group:	• Overweight:
Caucasian (%)	• Obese:
Attendance to any other weight loss program (%)	o 30-34
Education (%):	o 35-39
	o >40
• High school	Waist circumference (mean ±SD)
• Post-Secondary school	Blood Pressure (mean ±SD)
Income (%):	• Systolic BP
• <$15,000	• Diastolic BP
• $15,000-$29,999	HbA1c (mean ±SD)
• $30000-$49,999	Type II Diabetes (%)
• $50000-$79,999	Hypertensive (%)
• >$80,000	Depression (%)
	Other co-morbidity (%)
	PACIC score (mean ±SD)
Distance to practice (mean±SD)	SF-12 (mean ±SD)
	EQ5D (mean ±SD)

Patient assessment includes: baseline demographics and chronic disease presence (Table 3), measures of self-reported quality of life (EQ5D, SF-12), for follow-up visits a survey on weight management as a chronic disease (modified PACIC, self-report of change behaviour, *i.e*. gym participation, external weight loss programs, number of visits to a healthcare provider for weight management), and measurement of resting heart rate, blood pressure, and basic anthropometric measurements (including height, weight, waist circumferences). We will also monitor compliance with recommended laboratory studies (HbA1c for those with diabetes q 6 months, and for patients over 40 years, fasting cholesterol panel and glucose). If patients are unable to participate in the follow-up in person, a telephone option will be offered. The in-person visit will take 30 minutes per visit on up to five occasions.

### Quantitative data analysis: patient study

Demographic and health variables (Table 3) will be compared using either *t*-test for continuous variables or chi-square test for categorical variables. Main outcome measures are SF-12 and change in weight and BMI. Practice level clustering effects on the secondary outcomes, multilevel models (random effects model) will be considered. Adjustments will be made for individual level characteristics, the random factor (intervention or control practice), nurse and other practice level characteristics. Changes at follow-up will be analyzed using multilevel models with the baseline values as a covariate and to handle missing data [23]. Data modeling is hypothesis-led rather than data-driven, hence all analysis are predetermined. STATA 12 (StataCorp, TX, USA) will be used for statistical analyses.

### Selection of participants for qualitative patient sub-study

At 12 months, a sample of patients who agreed to be contacted will be selected and consented for individual semi-structured interviews. This will address patient-specific experiences of PCN weight management efforts. Purposive sampling will deliberately seek out a wide range of individuals. We will use a pragmatic approach, sampling until thematic saturation is reached. Selection factors will include 5AsT versus control practice affiliation, weight loss success, comorbid medical conditions, and PCN program attendance. Results of the quantitative and qualitative data merge will determine the focus and extent of patient interviews and sub-analyses.

### Qualitative data handling and analysis

Patient interviews will be handled and analyzed using the same techniques and tools as the provider study. The codebook created for the provider study will influence transcript coding.

### Trial status

The 5AsT has been approved by the University of Alberta ethics board and has been registered at Trials.gov (NCT01967797). It is funded by an Alberta Innovates Health Solutions grant.

## Discussion

The 5AsT trial is a theoretically informed, pragmatic trial that uses a multi-level collaborative approach to aim to sustainably change practitioner behaviors to improve obesity management in primary care.

This project is grounded in established theoretical frameworks for behavior change and complex innovations and leverages bi-directional knowledge translation between clinical and academic partners to comprehensively evaluate the implementation of the 5AsT practice change intervention.

Evaluation guided by the RE-AIM framework is particularly useful for determining programs that work in real-world environments [13]. By widening the evaluative focus beyond efficacy, the overall suitability and investment-to-results assessment of the intervention can be made.

As suggested in the conceptual framework of complex innovation implementation Complex Innovations, process is distinct from the evaluation of the 5AsT providers’ behaviour change [11]. We will use a rigorous mixed-methods study design to distinguish issues with implementation process from effectiveness of the intervention. Maximal systemic impact of the research is attained by implementing an integrated approach for sustainability at the outset of the project [24]. Sustainability will be achieved by leveraging existing clinical resources and infrastructure. We will continue to monitor the primary outcome measure for another year post the intervention phase of the project.

### Main findings/messages

This research proposal uses a pragmatic design particularly suited for evaluating the complex, real-world interventions typical of primary care settings [13]. Pragmatic trials measure effectiveness (*i.e*., the degree of beneficial effect in real clinical practice) and are conducted on participants who represent the full spectrum of the population to which the treatment might be applied. It is important to extensively describe the context and population in detail. In addition to the complexity of this research setting, our target patient population (patients who are overweight or obese) constitute one additional level of complexity, given the large variability in the etiology, comorbidity, and drivers of obesity as well as the variable compliance, readiness to change, and treatment preferences. Nevertheless, a key methodological issue in pragmatic trials is finding the right balance between external and internal validity [25]. Provider behaviour is a key feature in any primary care intervention. If providers do not have the skills, beliefs and confidence to be able to intervene effectively with patients, there will not be an improvement in obesity management in primary care. The 5AsT trial aims to understand what provider factors are instrumental to increase the quality of obesity management in primary care.

### Strengths

The strengths of this study are that it is a pragmatic intervention conducted in a real-world setting of a large and diverse Primary Care Network. The mixed methods study design will provide contextual insights into the intervention process and the outcomes. The bidirectional nature of the design of the intervention will ensure relevance to practitioners.

### Limitations

The pragmatic nature of the study design limits the ability to restrict or steer patients’ access to programming within the system, resulting in possible imbalance between practice contributions to the patient cohort. Furthermore, the dynamic and evolving clinical environment may result in shifting context and priorities within the network over time. Generalizability to other primary care networks and practitioners may require further adaption and intervention strategies tailored to those settings.

### Summary

The 5AsT trial addresses a need for knowledge exchange around obesity management in primary care in a practical and sustainable format geared towards real-life situations. The use of existing resources, collaborative design, practice facilitation, and integrated feedback loops cultivate an applicable, repeatable and adaptable approach to increasing the quality and quantity of primary care weight management visits. Its mixed method design will provide rich material to evaluate intervention effectiveness. A comprehensive 5AsT intervention implementation plan will address identified key barriers to obesity management in primary care.

## Abbreviations

CON: Canadian Obesity Network – Réseau Canadien en Obésité (CON-RCO); FTE: Fraction of a full time (1.0) appointment held by a practitioner (*i.e*., 0.5 is half time); Panel: Number of patients affiliated with a primary care practice; PCN: Primary care network; RN: Registered nurse; ESPCN: Edmonton south side primary care network.

## Competing interests

The 5As Team Study is funded by an Alberta Innovates Health Solutions CRIO Project Grant, with significant in kind support from the Edmonton South Side Primary Care Network.

Drs. Campbell-Scherer, Asselin, Osunlana, Rueda-Clausen, Johnson, A.A. Ogunleye, Manca have nothing to disclose. Sheri Fielding and Robin Anderson have nothing to disclose.

Dr. Cave reports grants from Alberta Innovates Health Solutions, during the conduct of the study.

Dr. Sharma is a member of an Advisory Board or equivalent with a commercial organization (Vivus: Consultancy for anti-obesity drug; Novo Nordisk: National [Canada] Advisory Board for anti-diabetes drug; Boeinger-Ingelheim: National and International Advisory Boards for Anti-hypertension and anti-diabetes drug).

Dr. Sharma is a member of a Speakers bureau (Vivus: Payment for development of educational presentations including service on speaker’ bureau).

## Authors’ contributions

DCS, CRC, AS conceived and designed the study with support from JJ, DM, SF, RA, and AC. DCS, AOs, and JA refined the protocol. AOg wrote the quantitative analysis plan. DCS, JA, AS drafted this manuscript with all authors providing critical comments and revisions. All authors have read and approved the final version.
